# Editorial: MEDICIS-promed: Advances in radioactive ion beams for nuclear medicine

**DOI:** 10.3389/fmed.2022.1013619

**Published:** 2022-10-03

**Authors:** Thierry Stora, John O. Prior, Clemens Decristoforo

**Affiliations:** ^1^European Organization for Nuclear Research (CERN), Geneva, Switzerland; ^2^Nuclear Medicine and Molecular Imaging Department, Lausanne University Hospital, Lausanne, Switzerland; ^3^Faculty of Biology and Medicine, University of Lausanne, Lausanne, Switzerland; ^4^Department of Nuclear Medicine, Medical University Innsbruck, Innsbruck, Austria

**Keywords:** radioisotopes, radiopharmaceutical, nuclear medicine, theranostics, CERN, MEDICIS, mass separation, radioactive beams

This special issue highlights advances in the availability of radionuclides for medical application based on developments of the production methods using ion beam and separation technologies. MEDICIS-Promed, a project funded within the H2020 framework program of the European Commission, aimed to train young scientists to provide the basis for novel production methods and for systems for personalized medicine, combining functional imaging and treatments based on radioactive ion beam mass-separation.

MEDICIS is an extension of the ISOLDE class A laboratory at CERN. It is a facility dedicated to the production of radionuclides for research in the medical field. It comprises an irradiation station located in the beam dump of the HRS target station, a remote handling system, an isotope mass separation system and a simple radiochemistry laboratory (Figure 1).

**Figure 1:**
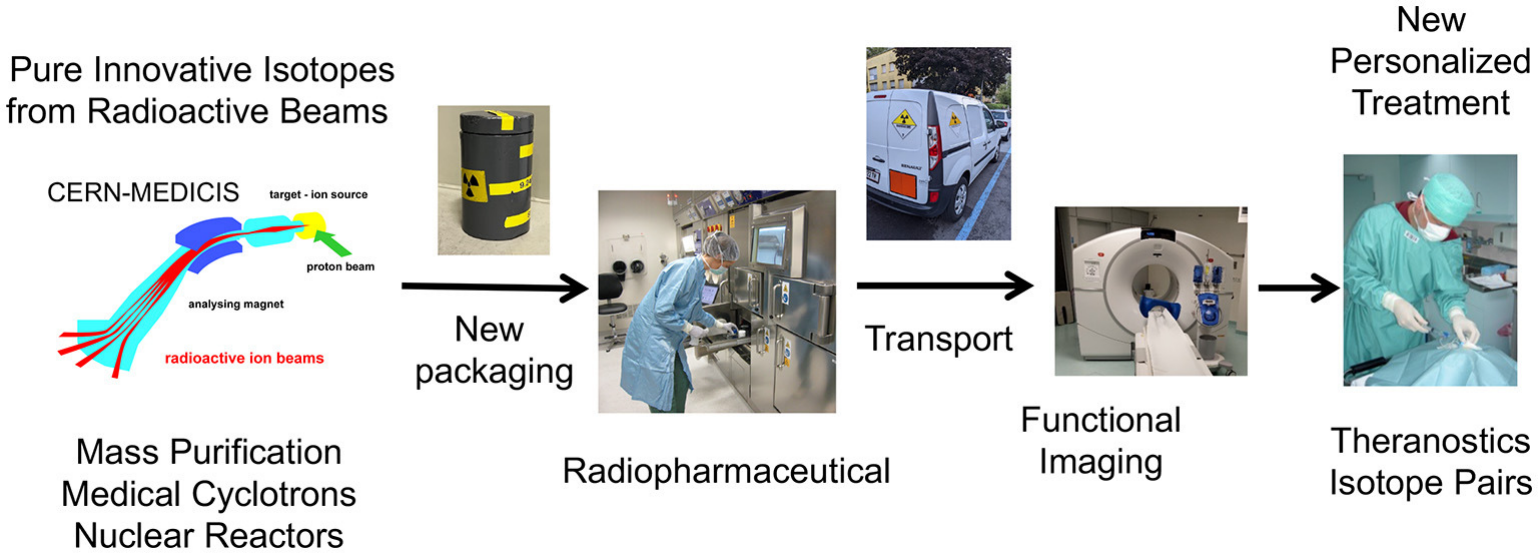
MEDICIS-Promed overview of innovative treatments based on radioactive ion beams: from production, transport to preclinical and clinical studies.

In Martinez Palenzuela et al., details of the MEDICIS beam lines are provided. It consists of a simple target station and related beam optics, along with a slit system and an isotope collection system. The required modifications to operate a laser ion source with a (laser-) beam window and impact of the modified magnetic field homogeneity are described. Operated with 1+ ion sources suitable for Radioactive Ion Beams used in ISOLDE, a mass resolving power dm/m~400 is simulated and experimentally verified with a VADIS ion source, allowing to achieve enrichment factors of x1,000–10,000 as shown with subsequent examples of this topical Frontiers article, enabling the production of radionuclides with high molar activities, e.g., Tb, Sm, or Er, that was not possible until then.

In Duchemin, Ramos et al., an overview of the operation of MEDICIS as a collaboration with external institutes is provided, starting with a 1st Radioactive Ion Beam commissioned in 2017. The operation of the facility with proton irradiations in 2018, with external sources provided by neutron irradiation in reactors and proton irradiation in cyclotrons during 2019–2020 in LS2 is described. The separation of small batches of radionuclides implanted in gold foils coated with zinc allowed to show the potential of the facility and collaboration—providing non-conventional radionuclides both for diagnostic and therapeutic applications. More specifically, radio-lanthanides were produced, as well as alpha emitting and Auger emitting radionuclides such as ^225^Ac and ^165^Er. They were shipped across Europe, including UK partners such as NPL and researchers in Lisbon, depending on the half-lives of the radionuclide under consideration. ^149^Tb, with its ~4 h half-life, must follow similar logistics constraints as ^18^F and was shipped to the neighboring CHUV research groups in Lausanne.

The newly started MELISSA laser ion source allowed to extend the scope and the efficiency of the radionuclide beams separated from different targets. With its element specific scheme developments, it reached high efficiency with stable elements such as Er, Tb, Yb, Ac as shown in Gadelshin et al.. Reaching 50% and more, it matches the best efficiencies achieved to date for radioelements investigated in facilities using the ISOL method, demonstrating the proper implementation of the new MELISSA source with 2 Ti:Sa lasers used in combination.

A few examples are provided by Heinke et al. and Talip et al., in which the target irradiation and later the separation performances are shown for ^167^Tm and ^169^Er. ^169^Er is a well-known radionuclide applied in nuclear medicine for radiosynovectomy. However, its scope of application had been limited because of its very low specific activity, preventing its use in targeted molecular therapy and in the field of theranostics.

^153^Sm by Van Voorde et al. is another example for which its application in nuclear medicine is restricted to pain relief treatments used for bone metastases of advanced stage prostate cancer. Its high molar activity grade made available by mass separation has allowed the synthesis of radiopharmaceuticals such as ^153^Sm-DOTA-HSA in a proof-of-concept experiment, paving the way toward the application of this radionuclide with suitable half-life, imaging properties as well as therapeutic properties to be tested and compared to, e.g., well-known ^177^Lu-PSMA radiobioconjugates presently investigated in several clinical trials.

The production and related cross-sections of terbium radionuclides with high energy proton beams on tantalum targets and of ^67^Cu with deuteron beams on ^70^Zn targets is presented by Nigron et al. and Duchemin, Cocolios et al., respectively. High energy beams and targets with limited enrichment factors lead to the production of radionuclidic impurities which are quantified there to find the best parameters to reach high activity, purity, and manage the required associated chemical or mass separation steps.

Penescu et al. extends the scope of the present topical Frontier issue to external radiotherapy, considering the diagnostics potential of PET-emitting ^11^C ions for treatment and dose distribution monitoring. While the success of treatments with Carbon-ions relies on the proper delivery of the dose to targeted tumors while preserving organs at risk, this paper reviews the requirements for facilities able to combine a ^11^C source with present-day operating synchrotron treatment facilities such as CNAO or MEDAUSTRON. In particular, the coupling of a medical cyclotron to such facility allows to develop the appropriate acceleration scheme with high efficiencies required to translate the cyclotron produced batches of ^11^C toward treatment and diagnostics hadron pulses.

In advancing applications derived from novel radionuclide production approaches there are essential steps also in the development of novel radiopharmaceuticals toward their clinical application. This covers aspects of modifying suitable targeting molecules for radiolabelling. In an interesting approach D'Onofrio et al. described the evaluation of a DOTA-Tetrazine, and its radiolabelling with trivalent metals, exemplified by ^90^Y and ^111^In. Their compound allows the application for pre-targeting approaches which have found applications especially in the context of using antibodies for targeting.

Optimization of radiolabelling procedures is also an important step in the development of novel radiopharmaceuticals, especially when sensitive targeting molecules are involved. Many trivalent radiometals included in this special issue require heating steps for conventional radiolabelling approaches which are not compatible with many targeting molecules. Cassells et al. describe radiolabelling experiments with ^161^Tb, using albumin as a model of a heat sensitive molecule and evaluated their stability *in vitro*, finally proposing DOTA, DOTAGA, and NETA derivatives as being suitable for terbium-radioisotopes.

Besides extensive testing of target interaction of novel radiopharmaceuticals, it is also essential in particular in the context of novel approaches in Theranostics to consider the biological effects in relation to the specific radiation emitted. In this context Pouget and Constanzo provided a mini review of revisiting the radiobiology of targeted alpha therapy. Even though it is recognized the alpha particles are highly cytotoxic producing complex DNA lesions and therefore the cell nucleus is seen as the primary target, recent research has shown that this paradigm is no longer valid and that alpha therapies are also effective in larger tumors or when a ligand carrying the alpha-emitter only binds to the cell surface. Also, bystander effects and immunological responses are increasingly recognized to play an important role for radionuclide therapy approaches, which is excellently summarized in this review. This, however, not only holds true for alpha emitters but also for other novel radionuclides emitting low energy electrons (e.g., Auger emitters).

Even if a new radiopharmaceutical has been developed and characterized preclinically, also logistics should not be forgotten, in particular considering the shipment of highly radioactive materials both in the context of the radiopharmaceutical preparation, but also for the clinical application. In the case of novel radionuclides there are limitations due to conservative radiation transport safety approaches when no coefficients are available to calculate the appropriate shipment category. In their paper Maietta et al. use Monte Carlo simulations for novel terbium radioisotopes, which can be expanded to other radionuclides, and allows simulation doses in different exposure scenarios, from which relevant coefficients can be derived.

In the process of the development of a novel radiopharmaceutical, the clinical translation process requires compliance with regulations not only related to radiation safety, but also from pharmaceutical legislation. Decristoforo et al. discuss the main regulatory framework for novel radionuclides and the hurdles involved. They identify the limitations in the current legislation, in particular in relation to the legal definitions of radionuclides for pharmaceutical applications. Within the PRISMAP project the requirements for pharmaceutical standardization and harmonization in the context of developing radiopharmaceuticals using novel radionuclides, including those derived from new ion beam applications, have been summarized and specified (1), and can guide this development process to the clinical application.

Closer to clinical application, Burkhardt et al. present a mini-review of potential medical application of novel radioisotopes to treat pancreatic cancer. Within the CERN MEDICIS collaboration, the authors present a non-exhaustive list of potential applications of the wide isotope production of this facility. These radioisotopes can potentially be used for targeted application (using neurotensin or somatostatin receptors, radioimmunotherapy, fibroblast activation protein [FAP] or FAP-specific enzyme inhibitors [FAPI], and integrin-based antibodies) or brachytherapy applications (CT-guided percutaneous implantation, endoscopic ultrasonography-guided, or robotic minimally invasive implanted).

Finally, Naskar and Lahiri present the theranostic quadruplet of terbium radionuclides (^152^Tb and ^155^Tb for diagnostics and ^149^Tb and ^161^Tb for therapy) which have promising applications, provided they can be produced in suitable quantities and adequate purity. The authors describe production pathways and show the role of CERN MEDICIS with spallation technique coupled with mass separation as a possible way to contribute to this endeavor.

Overall, this special issue on Advances in Radioactive Ion Beams for Nuclear Medicine provides an excellent overview with examples from different aspects of research areas involved in the translation of novel radionuclide production methods to clinical applications in Nuclear Medicine.

## Author contributions

TS, CD, and JP wrote the first article draft and revised the article. All authors contributed to the article and agreed with the last version to be accountable for the content of the work.

## Funding

The authors acknowledge the financial support of the E.U. through the MEDICIS-Promed program (grant agreement No. 642889) and the PRISMAP program (grant agreement No. 101008571).

## Conflict of interest

The authors declare that the research was conducted in the absence of any commercial or financial relationships that could be construed as a potential conflict of interest.

## Publisher's note

All claims expressed in this article are solely those of the authors and do not necessarily represent those of their affiliated organizations, or those of the publisher, the editors and the reviewers. Any product that may be evaluated in this article, or claim that may be made by its manufacturer, is not guaranteed or endorsed by the publisher.
